# Editorial: Gastrointestinal damage and metabolic disorders

**DOI:** 10.3389/fphys.2025.1648739

**Published:** 2025-06-30

**Authors:** Carolina Silva Schiebel, Samilla Santos Souza Mazeti, Marcelo Biondaro Gois, Daniele Maria-Ferreira

**Affiliations:** ^1^ Instituto de Pesquisa Pelé Pequeno Príncipe, Faculdades Pequeno Príncipe, Curitiba, Brazil; ^2^ Programa de Pós-graduação em Biotecnologia Aplicada à Saúde da Criança e do Adolescente, Faculdades Pequeno Príncipe, Curitiba, Brazil; ^3^ Faculdade de Ciências da Saúde, Universidade Federal de Rondonópolis, Rondonópolis, Brazil

**Keywords:** gastrointestinal damage, intestinal damage, gastrointestinal disorders, gut damage, gut dysbiosis, metabolic disorders

Gastrointestinal diseases are characterized by physiological and/or morphological changes in the gastrointestinal tract. These include impaired motility and integrity of the mucosa, dysregulated immune responses and an imbalance of the intestinal microbiota. Regardless of the specific underlying disease, these disorders can lead to significant clinical symptoms and contribute to the development of secondary pathologies. Ten articles have been published, which have been accessed more than 20,000 times.

In a review, Nie et al. emphasize the dysfunction of the gut-organ axis as a key factor in the impairment of the intestinal barrier. Extraintestinal organ dysfunction can exacerbate intestinal permeability through inflammatory responses, metabolic changes and altered intestinal perfusion, creating a feedback loop that exacerbates intestinal and systemic damage. Early intestinal barrier dysfunction may serve as a biomarker for extraintestinal disease and aid prognosis and timely intervention. They also summarized the key molecular mechanisms, including the role of TLR4/NF-κB/MAPK in inflammation, ApoM/S1P in vascular barrier dysfunction, WNT/β-catenin in stem cell regulation, and PI3K/Akt/mTOR in autophagy and protein synthesis. Finally, they discussed how microgravity increases intestinal permeability and alters tight junctions, microbiota composition, immune responses, and secretion of protective factors such as mucin and SIgA, suggesting a significant impact on intestinal homeostasis in the space environment.

Zhang et al. performed the first scientometric analysis of refractory gastroesophageal reflux disease. Using bibliometric methods, they identified key research trends focusing on standardized diagnostic and therapeutic approaches, underlying mechanisms, new surveillance techniques, and emerging pharmacological and procedural innovations. Their analysis highlighted neuroimmune interaction as a potentially crucial factor in the pathogenesis of gastroesophageal reflux disease, pointing to a promising direction for future mechanistic studies.

In a literature review, Yang et al. emphasized the therapeutic potential of Sijunzi decoction in the treatment of various gastrointestinal diseases. Clinical studies indicate its efficacy in functional dyspepsia, chronic gastritis, gastric cancer, irritable bowel syndrome, colon cancer and ulcerative colitis. These protective effects are associated with the regulation of gut microbiota, the attenuation of inflammation, the modulation of immune responses and the promotion of mucosal repair. However, the exact mechanisms underlying its effects are not yet fully understood.

Yeung conducted a literature review to track the development of infliximab use in Crohn’s disease. He identified key milestones, including the introduction of the CDAI in 1976, early evidence of infliximab efficacy in 1995, the first randomized trial in 1997, the ACCENT I and II trials, and the European consensus guidelines. He concluded that these studies provide a solid basis for further research.

Bi et al. investigated the role of miRNA dysregulation in chronic atrophic gastritis and found significant upregulation of miR-3613-5p in gastric mucosa and serum of gastric cancer patients as well as in mucosal tissue of patients with chronic atrophic gastritis, tumor samples and gastric cancer cell lines (via GEO database analysis). In mouse models, the expression of miR-3613-5p was also increased in the gastric mucosa. Functionally, its overexpression promoted gastric cancer cell proliferation and migration *in vitro*, while silencing attenuated gastric mucosal pathology. Mechanistically, miR-3613-5p directly interferes with the 3′UTR of AQP4 and suppresses its expression. The authors concluded that miR-3613-5p contributes to the progression of chronic atrophic gastritis to gastric cancer by downregulating AQP4 and could serve as a biomarker and therapeutic target.

Using *in vivo* (mouse model of intestinal I/R injury, including C57BL/6J and vitamin D receptor knockout mice) and *in vitro* approaches (IEC-6 cells with VDR and ATF4 knockdown), Zhang et al. demonstrated that paricalcitol is a potential therapeutic agent for intestinal I/R injury. The authors showed that paricalcitol activated VDR and suppressed the ATF4–CHOP signaling pathway, thereby reducing endoplasmic reticulum stress and apoptosis and attenuating I/R-induced intestinal injury *in vivo* and H/R-induced injury *in vitro*. Moreover, VDR deficiency exacerbated I/R injury, highlighting its important protective role for intestinal epithelial cells.

Canová et al. investigated the effects of celastrol on the galaninergic system in the heart and liver of male C57BL/6J mice with diet-induced obesity and MASLD/MASH. Using a high-fat Western diet model, they showed that Celastrol was safe and effectively reduced food and energy intake, body fat, liver weight and progression from MASLD to MASH. In addition, celastrol had a positive effect on the galaninergic system, suggesting its potential as a therapeutic option for obesity and related metabolic disorders.

Guo et al. investigated the association between glucagon-like peptide-1 receptor agonists (GLP-1 RAs) and acute pancreatitis (AP) using data from the FDA Adverse Event Reporting System (FAERS) and a case series of 39 patients. Analysis of reports from January 2005 through September 2023 identified a total of 6,751 cases of suspected AP associated with GLP-1 RAs, with an average patient age of 57 years and 98.3% classified as severe. Signal detection analysis (ROR, PRR, BCPNN, MGPS) revealed a positive association between AP and all GLP-1 RAs examined, with stronger signals for exenatide and liraglutide. These results suggest that clinical vigilance is required when prescribing GLP-1 RAs given the potential risk of this serious side effect.

Sun et al. investigated the role of carbamoyl phosphate synthetase 1 (CPS1), a rate-limiting enzyme in the urea cycle, in the development *versus* persistence of glucagon-induced hyperglycemia using *in vivo*, *in vitro* and *in silico* approaches. CPS1 played a key role in mediating glucagon-induced hepatic gluconeogenesis. In addition, they showed that cynarin could be a natural inhibitor of CPS1 and has the potential of a therapeutic agent for the treatment of diabetes.

Finally, Łukawska and Mulak investigated the relationship between FGF21 serum levels, inflammatory markers and indicators of nutritional status in patients with inflammatory bowel disease (IBD). The severity of intestinal inflammation is associated with elevated FGF21 levels, which correlate negatively with indicators of nutritional status. These results suggest that dysregulation of FGF21 secretion may contribute to the multifactorial pathogenesis of malnutrition and weight loss in IBD patients.

This Research Topic has produced new and relevant findings that significantly advance current understanding in this field. We would like to thank all authors, reviewers and editors for their valuable contributions to the development and dissemination of this Research Topic.

